# Doctor's Wills

**Published:** 1913-11-01

**Authors:** 


### Doctor's Wills.

Fleet-Surgeon Arthur Willoughby Russell, R.N. (retired), of Kingstown, Dublin, has left personal estate in the United Kingdom valued at £8,763.

Dr. Edward James Nix, M.D., of Portland Place, W., physician to the All Saints' Children's Hospital, Margaret Street, and to the St. Elizabeth Home for Incurables, Mortimer Street, has left estate of which the net personalty has been sworn at £23,032.

